# Referring to multimodal rehabilitation for patients with musculoskeletal disorders – a register study in primary health care

**DOI:** 10.1186/s12913-016-1948-7

**Published:** 2017-01-07

**Authors:** Charlotte Post Sennehed, Sara Holmberg, Kjerstin Stigmar, Malin Forsbrand, Ingemar F. Petersson, Anja Nyberg, Birgitta Grahn

**Affiliations:** 1 Faculty of Medicine, Department of Clinical Sciences Lund, Orthopedics, Lund University, Lund, Sweden; 2 Epidemiology and Register Centre South, Region Skåne, Lund, Sweden; 3 Department of Research and Development, Region Kronoberg, Växjö, Sweden; 4 Division of Occupational and Environmental Medicine, Institute of Laboratory Medicine, Lund University, Lund, Sweden; 5 Department of Health Sciences, Physiotherapy, Lund University, Lund, Sweden; 6 Blekinge Centre of Competence, Karlskrona, Sweden; 7 Skåne Regional Council, Region Skåne, Department of Healthcare Governance, Malmö, Sweden

**Keywords:** Multiprofessional rehabilitation, Musculoskeletal disorders, Primary health care, Referral rate

## Abstract

**Background:**

In 2008, the Swedish government introduced a National Rehabilitation Program, in which the government financially reimburses the county councils for evidence-based multimodal rehabilitation (MMR) interventions. The target group is patients of working age with musculoskeletal disorders (MSD), expected to return to work or remain at work after rehabilitation. Much attention in the evaluations has been on patient outcomes and on processes. We lack knowledge about how factors related to health care providers and community can have an impact on how patients have access to MMR. The aim of this study was therefore to study the impact of health care provider and community related factors on referrals to MMR in patients with MSD applying for health care in primary health care.

**Methods:**

This was a primary health care-based cohort study based on prospectively ascertained register data. All primary health care centres (PHCC) contracted in Region Skåne in 2010-2012, referring to MMR were included (*n* = 153). The health care provider factors studied were: community size, PHCC size, public or private PHCC, whether or not the PHCCs provided their own MMR, burden of illness and the community socioeconomic status among the registered population at the PHCCs. The results are presented with descriptive statistics and for the analysis, non-parametric and multiple linear regression analyses were applied.

**Results:**

PHCCs located in larger communities sent more referrals/1000 registered population (*p* = 0.020). Private PHCCs sent more referrals/1000 registered population compared to public units (*p* = 0.035). Factors related to more MMR referrals/1000 registered population in the multiple regression analyses were PHCCs located in medium and large communities and with above average socioeconomic status among the registered population at the PHCCs, private PHCC and PHCCs providing their own MMR. The explanation degree for the final model was 24.5%.

**Conclusions:**

We found that referral rates to MMR were positively associated with PHCCs located in medium and large sized communities with higher socioeconomic status among the registered population, private PHCCs and PHCCs providing their own MMR. Patients with MSD are thus facing significant inequities and were thus not offered the same opportunities for referrals to rehabilitation regardless of which PHCC they visited.

## Background

Musculoskeletal disorders (MSD) are among the most common reasons for sick leave in western countries [1–3]. The Global Burden of Disease Study reported in 2012, that MSD caused 21% of all years lived with disability and within MSD, the largest group was those with low back pain [4]. In Sweden MSD encompasses the second largest proportion of sick leave. In 2012, 25% of those on sick leave were so due to these disorders (personal communication, The Swedish Social Insurance Agency) and incidence and costs are increasing [5]. For reincentivizing, i.e. stay at work and return to work, the patient needs strategies to regain physical, mental and social functions [6]. Most patients in Sweden with MSD get their first treatment in primary health care (PHC) and when patients need they can receive further rehabilitation by referral from the primary health care centres (PHCC) to secondary care. In 2012, about 20-30% of the total numbers of visits in PHC were made by patients with MSD [7] and patients diagnosed with back pain consumed twice as much health care resource as the general population [8].

In 2008, the Swedish government introduced a warranty National Rehabilitation Program with the ambition to provide all inhabitants in Sweden with evidence-based rehabilitation in PHC [9]. Different specialized rehabilitation programmes for long-lasting pain have been evaluated and there is some evidence that multimodal rehabilitation (MMR) is effective in relation to return to work [10, 11] and also cost effectiveness has been proven [12, 13]. When rehabilitation has been combined with work place interventions MMR has been found effective [14, 15].

The state financially reimburses the PHC in the county councils for this evidence-based rehabilitation directed to patients in working age, 16-67 years of age, with mild to moderate mental disorders and patients with MSD, mainly neck, shoulder and back pain [16]. The National Rehabilitation program is proposed to strengthen the opportunities for rehabilitation for the two large patient groups at risk for developing long-lasting problems and sick leave [17] and is intended to improve function, work ability and to reduce social costs due to ill health and sick leave.

In Sweden, patients with MSD are offered MMR after referrals in PHC. The rehabilitation can be provided by private or public contracted units all funded by the county councils. To get a referral to MMR the patient has to visit a physician in PHC for medical assessment. Thus it is not possible for the patient to access MMR without referral. MMR involves a multiprofessional team with physician, physiotherapist, psychologist and occupational therapist. MMR is given full or part-time over four to eight weeks and includes cognitive behavioural therapy, physical therapy and patient education. The rehabilitation is mainly provided as group treatments. Therefore it is not possible to get only individual treatment within the MMR programme. For each completed MMR the unit executing the care receives financial compensation. During the first years of the National Rehabilitation Program the county councils was compensated with 45 000 SEK (5473 USD) per patient.

The MMR within the National Rehabilitation Program implemented in Swedish PHC has been evaluated with special attention on process, implementation and development. The implementation process has been slow, partly due to ambivalence regarding development of new treatment modalities, organisational uncertainties and ambiguities concerning patient selection [18]. However, the evaluation found that PHCCs experienced opportunities for expanded operations and were positive towards a focus on psychosocial interventions for patients [19, 20]. Difficulties in implementation were seen, e.g. that MMR teams had inadequate competence concerning rehabilitation focusing work ability and sick-listed patients return to work [18]. In summary, there is lack of evidence regarding early rehabilitation in PHC for patients with MSD [21].

Much attention in the evaluations of the National Rehabilitation Program has been on patient outcomes [22] and on process [10, 20]. We know from other contexts, for example regarding use of diagnostic methods and antibiotic treatment, that there are big inexplicable differences between caregivers and geographical areas [23, 24]. In order to prevent inequity there is a need to know more about health care provider factors (organisational and economic) in relation to referrals and health outcomes. To our knowledge, this is the first time health care provider factors are studied in relation to referrals to MMR. The aim of this study was therefore to study the impact of health care provider and community related factors on referrals to MMR in patients with MSD applying for health care in PHC.

## Methods

### Design

This was a PHC-based cohort study based on prospectively ascertained register data from the Healthcare Governance in Region Skåne in south Sweden.

### Setting

Almost all health care in Sweden is tax-financed. Both public and private health care providers are available. PHC is mostly the first care giver instance in Sweden, responsible for all basic healthcare and referrals to appropriate specialist healthcare. Region Skåne has 13% of Sweden’s population and increased from 1 243 329 inhabitants in year 2010 to 1 263 088 inhabitants in year 2012. Since 2010, all inhabitants in Region Skåne have opportunities to actively choose any public or private accredited PHCC for registration. Patients who do not actively choose a PHCC are registered based on the latest visit or the shortest distance to the PHCC. The PHCCs need to be accredited by the local county council and the reimbursement paid to the PHCC follows the patient. The PHCCs are obligated to participate in follow-ups on quality of care. Measurements of burden of illness and socioeconomic status of the registered population determine the financial compensation for the PHCCs. In Region Skåne, as well in Sweden in general, PHCCs are multiprofessional with physician, nurse, physiotherapist, occupational therapist and psychologist [25].

### Data collection

We obtained permission to use available data from the Healthcare Governance in Region Skåne. No questionnaires were sent to PHC or PHCCs in order to obtain their opinions. Inclusion criteria were all accredited PHCCs contracted in Region Skåne, who had issued any referrals during 2010-2012. We identified 233 health care units in the years 2010 to 2012 that had issued referrals to MMR in PHC.

Number of inhabitants in the community (community size) where the PHCC was located was retrieved from Statistics Sweden. From the Healthcare Governance in Region Skåne we assessed data about number of referrals/PHCC, registered population/unit (PHCC size), model of health care (public or private PHCC), whether or not the PHCC provided their own MMR team (internal/external MMR) burden of illness/burden of morbidity (ACG) and socioeconomic status (CNI).

Data from the Regional Council Skåne and Statistics Sweden were input manually into a SPSS 20.0 database for analysis. Data quality was thoroughly checked and validated.

**Fig. 1:**
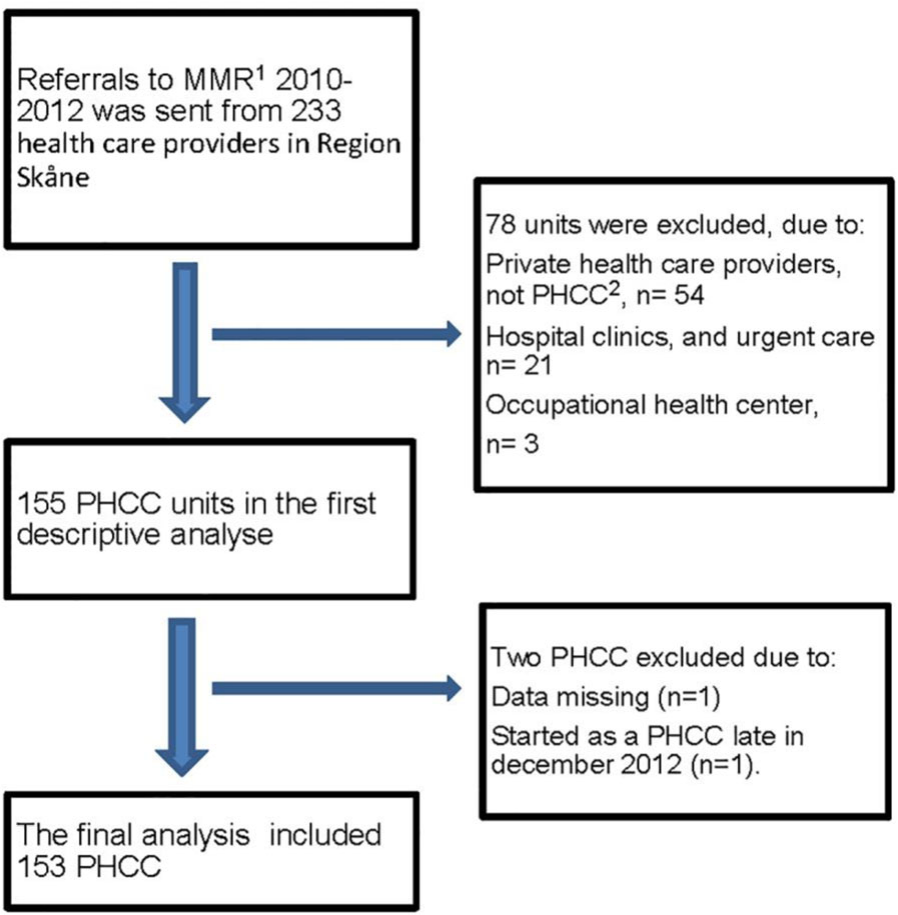
Flowchart of inclusion and exclusion of the health care providers in Region Skåne, Sweden. ^1^Multimodal Rehabilitation. ^2^Primary Health Care Center

Excluded units were clinics in specialist care such as psychiatry, occupational health and individual health care providers not incorporated in the PHC organisation and hence not accredited. The majority of these excluded units had only occasional referrals to MMR over the three years and the referrals were returned to remittance. Another two units were excluded due to missing data and starting up the unit in late 2012, just a few days before the inclusion was closed. The final analysis therefore included 153 PHCCs (Fig. 1).

### Outcome

The outcome was number of referrals to MMR/1000 registered in the population at the PHCC (referral rate).

### Independent variables

Independent variables operationalising health care provider factors and community were chosen based on clinical and organisational experience, in conjunction with previous research findings and limitations.

The accessibility of health care services varies with geographical location, due to distances but also due to fewer options to choose care providers in less populated areas [26–28]. In Sweden fewer new PHCCs are established in areas with lower socio-economic status [26]. Geographical related access to PHC might therefore impact referral to MMR [26]. Studies have shown that PHCCs size may affect access to health care and how patients experience the quality of healthcare regarding accessibility of care, consultation length and continuity [29, 30]. Smaller PHCCs have better accessibility and better continuity of care compared with larger units. In a Swedish report the proportion of various referrals per PHCC varied with unit size [30]. New PHCCs in Sweden are almost exclusively privately operated and are established primarily in urban areas [26]. The introduction of freedom of choice in PHC has shifted care to become more customer-oriented [31]. Since patients attending private units generally have better health, [32, 33] there is a risk of inequity. Therefore the organisational model of health care such as the PHCCs providing their own MMR might impact referrals. The reimbursement to the PHCCs follows the patient and the PHCCs need to finance their operations [26]. To provide in-depth rehabilitation, such as MMR, can provide great additional income for the PHCC [19, 20]. This might be a financial incentive for more referrals.

Burden of illness/burden of morbidity is based on Adjusted Clinical Groups (ACG) and was developed by the School of Hygiene and Public Health at John Hopkins University in Baltimore, USA [34, 35]. ACG can be a useful instrument to describe the disease patterns, age and gender in a population [36]. For each PHCC a weighted ACG value is calculated based on recorded diagnoses among its patients and used to estimate the financial compensation to each PHCC. The mean value is 1.0. Values above 1.0 indicate increased morbidity and health care burden. This is important as the patients’ type of illness, degree of illness and morbidity at the PHCC might affect how referrals are written.

Socioeconomic status among the registered population at the PHCCs is based on Care Need Index (CNI), which assess health risk indicators for populations [37]. CNI includes the following criteria: elderly persons living alone, children under age 5, unemployed, low education, single parents, recently moved and born in another country. For each PHCC a weighted CNI is calculated based on its registered population and is used to additionally calculate the financial compensation to each PHCC within the region [37, 38]. Values above 1.0 indicate a lower socioeconomic status and higher risk of developing illness in the defined population. Populations with higher socioeconomic status generally have better health and are more likely to request care [32, 33]. This might impact health professionals’ tendency to provide referrals.

### Statistical analysis

Descriptive statistical analyses for the three years were made by percentage, median and quartiles. In order to do statistical analysis, the independent variables were grouped according to criteria described below. The cut off values were decided using a pragmatic approach based on the a priori hypotheses in combination with the data generated. Concerning community size of PHCC location the variables was divided in relatively equal groups relevant to Swedish demography, very small (<10 000 inhabitants), small (10 000-34 999 inhabitants), medium (35 000-100 000 inhabitants) and large (>100 000 inhabitants) in the community. The variables concerning PHCC size were grouped as small (<6000 registered population), medium (6000-10 500 registered population) and large (>10 500 registered population). This division matches with Swedish standard and was relevant in the analysis. There were large variance regarding the PHCCs ACG (0.69-1.37) and CNI (0.53-2.44) values in line with previous studies [23, 30]. For the nonparametric tests and the multiple linear regressions analyses we needed to do sub-grouping, in order to do comparisons between higher and lower values. The value regarding burden of illness/ACG varied around 1.0. We categorized the units in three groups, PHCCs with lower (<0.95), medium (0.95-1.05) and higher (>1.05) burden of illness based on the distribution of ACG among the PHCCs. Socioeconomic status/CNI also varied around 1.0. We categorized the units in three groups, higher (<0.95), medium (0.95-1.05) and lower socioeconomic status (>1.05) based on the distribution of CNI among the PHCCs. Values below and above the medium groups indicate higher or lower burden of illness and higher or lower socioeconomic status, and therefore above or below normal. We used nonparametric tests to analyze the distribution of referral rate for each variable; PHCC location, PHCC size, whether or not the PHCC provided their own MMR, ACG and CNI. The Kruskal-Wallis test was used to analyze group differences for PHCCs location and size, ACG and CNI. The Mann-Whitney test was used when comparing public and private PHCCs and whether or not the PHCC provided their own MMR. Finally, multiple linear regression analyses were performed to find factors independently associated with referral rate/1000 registered population to MMR. In the nonparametric tests and the multiple linear regression analyses we used data from 2012. The reason for this was that MMR in Region Skåne was introduced in late 2009 and our intention was to study referrals to MMR after implementation had been stabilized. To achieve the final model, significant variables were provided from a stepwise procedure with *p* < 0.05 as inclusion criterion and *p* > 0.1 as the removal (of already included variables) criterion. Variables excluded by the stepwise regression model were PHCC size, ACG and the interaction term private PHCC and internal MMR. There were no other interaction terms identified. Since the independent variables were categorical unstandardized B were provided, as they directly indicate/represent the difference of referring/1000 inhabitants between one category/group and another. All variables were analyzed and finally community size where the PHCCs were located and socioeconomic status among the registered population at the PHCCs, private PHCCs and internal MMR at the PHCCs were put in the regression model. Constant was very small and small communities, small communities with lower socioeconomic status, public PHCCs and PHCCs with external MMR. *P*-values less than 0.05 were considered significant.

### Ethical considerations

This study has been approved by the Regional Ethical Review Board in Lund, Dnr 2014/290. In Region Skåne the PHCCs are obligated to participate in follow-ups on quality of care.We had agreements from the Regional Council Skåne to evaluate and study health care provider factors associated to referring to MMR.

## Results

PHCCs size varied with a registered population that ranged from 678 to 24 254. The care burden measured by ACG and CNI varied significantly between PHCCs (Table 1). A larger number of PHCCs were situated in very small communities with less than 10 000 inhabitants (38%) compared to large communities with more than 100 000 inhabitants (17%). Of the 153 PHCCs included, 89 (58%) were public units and 64 (42%) were private units. Thirty-seven (24%) PHCCs had internal MMR teams. Sixty-six PHCCs (45%) had a medium burden of illness (ACG value 0.95-1.05) and 89 PHCCs (60%) had higher socioeconomic status with low CNI value (<0.95) (Table 2). In 2010, five PHCCs did not send any referral to MMR. In 2011, two PHCCs did not send any referral to MMR and in 2012 four PHCCs did not send any referral to MMR. During the whole data collection period, two PHCCs were identified that had not sent any referrals to MMR. We excluded 78 units not defined as PHCCs; they were not incorporated in the PHC organisation and were not accredited.

**Table 1:** Characteristics of PHCCs^a^ in Region Skåne that had prescribed referrals to MMR^b^ in 2010-2012

	2010				2011				2012			
	n^f^	Md	Q1/Q3^e^	Min/Max	n^f^	Md	Q1/Q3^e^	Min/Max	n^f^	Md	Q1/Q3^e^	Min/Max
PHCCs size, registered population, total	126	7896	5456/11 306	790/21 456	148	7993	5418/11 118	724/24 254	148	8101	5690/10 962	678/21 861
Burden of illness, ACG^c^, Mean = 1.0 > 1.0 increased burden of illness	126	1.00	0.93/1.06	0.69–1.49	148	1.00	0.93/1.07	0.68–1.41	148	0.99	0.94/1.06	0.69–1.37
Socioeconomic status, CNI^d^, Mean = 1.0, > 1.0 worse socioeconomic status	126	1.03	0.90/1.20	0.57–2.01	148	1.00	0.89/1.17	0.59–2.51	148	0.91	0.83/1.07	0.53–2.44

^a^Primary health care center

^b^Multimodal rehabilitation

^c^Adjusted Clinical Groups

^d^Care Need Index

^e^Range for quartile 1 and quartile 3

^f^Missing PHCCs in year 2010 = 27, year 2011 = 5, year 2012 = 5

**Table 2:** Characteristics of PHCCs^a^ in Region Skåne that had prescribed referrals to MMR^b^ in 2012

	n^e^	%	Missing
PHCC location, size grouped			
Very small community < 10 000 inhabitants	58	38	
Small community 10 000-34 999 inhabitants	33	22	
Medium community 35 000-100 000 inhabitants	36	23	
Large community > 100 000 inhabitants	26	17	
PHCC size, registered population			
Small (<6 000)	44	29	5
Medium (6 000-10 500)	57	37	
Large (> 10 500)	47	31	
Model of Health Care			
Public PHCCs	89	58	
Private PHCCs	64	42	
PHCCs providing their own MMR			
Yes	37	24	
No	116	76	
Burden of illness, ACG^c^			
Mean = 1.0 > 1.0 increased burden of illness			
PHCCs with low ACG (<0.95)	42	28	
PHCCs with medium ACG (0.95-1.05)	66	45	
PHCCs with high ACG (>1.05)	40	27	
Socioeconomic status, CNI^d^			
Mean = 1.0, > 1.0 lower socioeconomic status			
PHCCs in community with low CNI (<0.95)	89	60	
PHCCs in community with medium CNI (0.95-1.05)	18	12	
PHCCs in community with high CNI (>1.05)	41	28	

^a^Primary Health Care Centre

^b^Multimodal rehabilitation

^c^Adjusted Clinical Groups

^d^Care Need Index

^e^Number of PHCCs

**Fig. 2:**
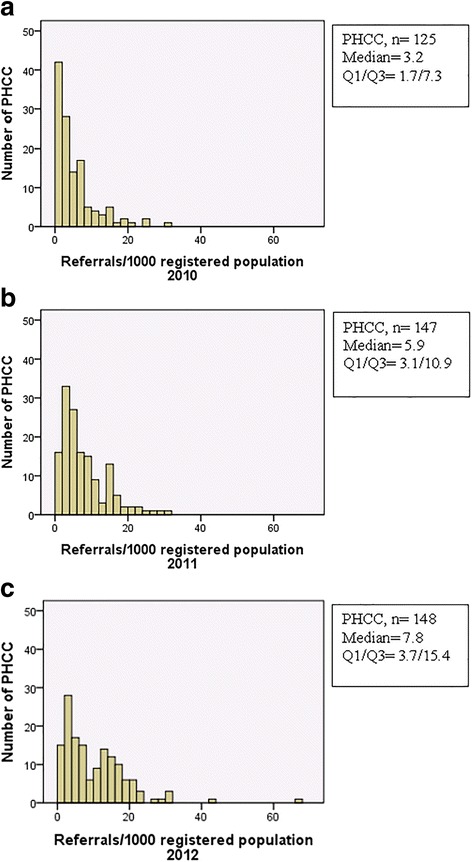
**a** Number of sent referrals/1000 registered population at the PHCCs in 2010. **b** Number of sent referrals/1000 registered population at the PHCCs in 2011. **c** Number of sent referrals/1000 registered population at the PHCCs in 2012

The total number of sent MMR referrals from all PHCCs (*n* = 153) included in the analysis increased from 6 078 referrals in 2010 to 13 153 sent referrals in 2012 (Table 3). Referral rate/1000 registered population increased from median 3.2 in 2010 to median 7.8 in 2012 (Table 3) and (Fig. 2a-c).

**Table 3:** Number of referrals to MMR^a^ from included PHCCs^b^ in year 2010-2012 in Region Skåne

	n^c^	Md^d^	Q1/Q3^e^	Min/Max
Referrals to MMR, total				
2010, 148 PHCC, missing = 5	6078	24	9/61	0–327
2011, 151 PHCC, missing = 2	9998	40	23/89	0–363
2012, 149 PHCC, missing = 4	13 153	65	29/117	1–383
Referrals to MMR/1000 registered population				
2010, 125 PHCC, missing = 28	675	3	2/7	0–31
2011, 147 PHCC, missing = 6	1150	6	3/11	0–30
2012, 148 PHCC, missing = 5	1538	8	4/15	0–66

^a^Multimodal rehabilitation

^b^Primary Health Care Center

^c^Referrals from included PHCCs

^d^Median, referrals from included PHCCs

^e^Range for quartile 1 and quartile 3

Nonparametric tests showed that PHCCs situated in larger communities sent more referrals/1000 registered population than those in smaller communities (*p* = 0.020) Private PHCCs sent more referrals/1000 registered population compared to public PHCCs, median 12.6 vs. 7.2 (*p* = 0.035) (Table 4).

**Table 4:** Number of referrals to MMR^a^/1000 registered population at the PHCCs^b^ according to health care provider factors in 2012

	Md^e^	Q1/Q3^f^	*p*
PHCC location, community size, grouped			0.020^g^
Referrals from very small community < 10 000 inhabitants, PHCCs *n* = 56 Referrals from small community 10 000-34 999 inhabitants, PHCCs *n* = 33 Referrals from medium community 35 000-100 000 inhabitants, PHCCs *n* = 35 Referrals from large community > 100 000 inhabitants, PHCCs *n* = 24	6.1 7.8 10.0 16.9	6.1/12.2 3.2/15.0 4.6/16.4 4.6/20.8	
PHCC size			0.614^g^
Referrals from small PHCCs (<6000 registered population), PHCCs *n* = 44 Referrals from medium PHCCs (6000-10 500 registered population), PHCCs *n* = 57 Referrals from large PHCCs (>10 500 registered population), PHCCs *n* = 47	6.5 8.1 9.2	3.3/15.4 4.4/14.4 3.5/15.8	
Model of health care, public or private PHCC			0.035^h^
Referrals from public PHCCs, PHCCs *n* = 86 Referrals from private PHCCs, PHCCs *n* = 62	7.2 12.6	3.8/13.0 12.6/17.6	
Referrals from PHCC providing its own MMR, yes or no			0.111^h^
Referrals from PHCCs providing their own MMR, no, PHCCs *n* = 112 Referrals from PHCCs providing their own MMR, yes, PHCCs *n* = 36	7.6 11.8	3.5/14.0 3.8/17.4	
Burden of illness, ACG^c^			0.476^g^
Mean = 1.0 > 1.0 increased burden of illness			
Referrals from PHCCs with low ACG (<0.95), PHCCs *n* = 42 Referrals from PHCCs with medium ACG (0.95-1.05,) PHCCs *n* = 66 Referrals from PHCCs with high ACG (>1.05), PHCCs *n* = 40	9.4 6.1 9.7	3.6/16.8 3.5/14.4 3.9/14.4	
Socioeconomic status, CNI^d^			
Mean = 1.0, > 1.0 worse socioeconomic status			0.322^g^
Referrals from PHCCs in community with low CNI (<0.95), PHCCs *n* = 89 Referrals from PHCCs in community with medium CNI (0.95-1.05), PHCCs *n* = 18 Referrals from PHCCs in community with high CNI (>1.05), PHCCs *n* = 41	7.6 13.8 7.2	3.8/14.8 4.2/18.7 3.3/15.5	

^a^Multimodal rehabilitation, ^b^Primary Health Care Center, ^c^Adjusted Clinical Groups, ^d^Care Need Index, ^e^Median, ^f^Range for quartile 1 and quartile 3, ^g^Kruskal-Wallis Test, ^h^Mann-Whitney U Test

The multiple linear regression analyses revealed that the referral rate was higher among PHCCs located in medium and large communities with higher socioeconomic status among the registered population. Referral rate was higher among private PHCCs and PHCCs with internal MMR regardless of community size and socioeconomic status (Table 5). The coefficient of determination for the final model was R^2^ = 24.5%.

**Table 5:** Health care provider factors independently related to referral rate to multimodal rehabilitation

		95% confidence interval for B		
Model^a^	B^c^	Lower bound	Upper bound	*p*
(Constant)^b^	5.36	3.26	7.46	<0.001
35-100 000 inhabitants with higher socioeconomic status	4.88	1.15	8.61	0.011
>100 000 inhabitants with higher socioeconomic status	16.98	10.44	23.51	<0.001
>100 000 inhabitants with lower socioeconomic status	7.29	3.26	11.31	<0.001
PHCC, private model of health care	3.95	1.33	6.57	0.003
PHCC, provides its own MMR	4.41	1.35	7.47	0.005

^a^Multiple regression analysis, R^2^ = 0.245

^b^Constant was very small and small communities, communities with 10 000-35 000 inhabitants with lower socioeconomic status, PHCC public model of health care and PHCC not providing its own MMR

^c^Unstandardized B coefficient

## Discussion

Patients with MSD are facing significant inequities and were thus not offered the same opportunities for referrals to MMR regardless of which PHCC they visited. Our analysis showed that health care provider related factors were significantly associated with referral rate to MMR in PHC. Sent referrals/1000 registered population to MMR was positively associated with PHCCs located in medium and large communities with higher socioeconomic status among the registered population, private PHCCs and PHCCs with internal MMR.

Previous research on MMR has focused on patient related factors, but one can expect that outcomes may also depend on factors related to the health care system. According to our results health care providing factors such as PHCC location, PHCC size, public or private PHCC, whether or not the PHCCs provided their own MMR treatment, the burden of illness and the socioeconomic status in the area are of importance for access to rehabilitation. Differences in attitudes to MMR might also be important, but this could not be captured by the present study design, since this study is based on register data only. The results shed light on factors related to referral rates to MMR and indicate that patients with MSD were not offered the same opportunities for rehabilitation depending on which PHCC they visited. If there are differences in who actually is offered MMR, this might have an impact also on patient outcomes after MMR.

The analysis revealed differences between public and private PHCCs in the referral rate to MMR. We believe that this could be related to private PHCCs having more experience and knowledge of MMR and which patients that would benefit from the treatment modality. Patients in private PHCCs might more frequently request MMR, or can be more motivated and/or have the resources to participate in MMR. Participation in the MMR requires motivation, the opportunity to be on sick leave and the ability to participate in theoretical parts. About 1100 PHCCs are accredited in Sweden and about 33% are private. There is a rapid change in Sweden with an increased number of private PHCCs, although tax financed. [25]. The reimbursement paid to PHCCs follows the patient and can be a factor of importance. A large number of registered patients and high flow of assessments might be an incentive to improve the economy of the PHCC. We have not found studies analyzing differences between private and public PHCCs and this is a future research of importance. Private PHCCs were in this study in many cases more recently established compared to public PHCCs and the registered population in private PHCCs had higher socioeconomic status compared to the registered population at public PHCCs [39]. Patients with higher socioeconomic status may have better prerequisites to choose among health care alternatives and also being able to choose among different rehabilitation alternatives. This is confirmed in an earlier report which focuses on Swedish patients' ability to register which PHCC they would like to be part of [40]. Women, highly educated and young people more often changed or considered to change PHCCs, while the less-educated and people living in smaller communities were less inclined to change PHCC [40]. Furthermore an unjustified difference between population groups often arises in the meeting between patients and health care professionals and therefore medical professionals have an important role of being aware of this and working towards a more equal health care [41].

Residential areas with lower socioeconomic status, in which patients may not visit the PHCC because of financial difficulties, might lead to differences in health care despite major illnesses in the patient groups. Another reason for differences in health care could be patients’ ability to influence the care given, propensity to make demands and ability to understand and process the information given [38, 42]. Large financial compensation for completed MMR has probably been a motivating factor for referring to MMR and this might also account for the higher referral rates among units with internal MMR. However, our results should be viewed with caution as the introduction of ACG and CNI to suit Swedish conditions has required a lot of redevelopment [43]. We had no access to diagnosis data and could not analyse number of referrals in relation to number of diagnosed registered population. Therefore the outcome referrals/1000 registered was chosen.

The study has been designed and undertaken, based on high quality data. A strong advantage is that the data covers an entire county council area. After meticulous checking of data we believe that all potential accredited PHCC in the area were included. Only two PHCCs not sending any referrals at all to MRR over the period were identified. Region Skåne has 13% of Sweden’s population and is in many ways representative for Sweden in terms of socio-demographic variables. As far as we know this is the first study about health care provider factors related to referral rate to MMR and we have not found any comparable analysis within PHC. We estimate that the study size is large enough to provide valid results. We found that PHCCs in the largest community and private PHCCs send more referrals/1000 registered population. This is in opposition to the Swedish model where everyone should be offered equivalent care [9]. In the future, health care providers need to be aware that all patients should have the same opportunities to be offered evidence based pain rehabilitation regardless of where they live, socioeconomic status and what PHCC they choose. The coefficient of determination for the final model was remarkable high (R^2^ = 24.5%). This high level is unusual in studies with this type of data, but in this study we found strong correlation between health care provider factors, community factors and referring rate to MMR.

In the nonparametric tests and the multiple linear regression analyses we used data from 2012 only. The reason for this was that MMR in Region Skåne was introduced in late 2009 and our intention was to study referrals to MMR after implementation had been stabilized, which can be seen in Tables 1 and 3. In 2012, the PHCCs had developed procedures and working methods, which also included screening instruments for identification of patients in need of MMR. The number of referrals from units without PHCC contracts decreased over time. In 2010, 52 units with no contracts sent 116 referrals and in 2012, eight units with no contracts sent 37 referrals. Implementation takes time and it is difficult organising new ways of working [18, 20, 44–46]. Difficulties in implementation and measurements are also confirmed in studies about organisation, work practices and evaluations in healthcare quality. Professionals motivation, values, behaviours and interactions with patients is a prerequisite to manage quality in health care [47]. Accounting for quality in healthcare can be problematic and can result in counterproductive effects when it is not fully understood [48]. Phillips et al [49] describes another measurement for health care organisations including interviews and observations to assess the quality in health care. Changes in the methods used and how work is organised in health care could lead to a better use of different competencies. Thereby professionals could focus on the right duties and areas within their competence [50].

The findings in our study can be helpful for different health care organisation, such as county councils, to ensure that health care is delivered and available for all inhabitants; in the end to prevent health inequity. However this study does not provide information on which type of rehabilitation is best for different patients’ needs. From a clinical perspective, equal care is not the same as equal rehabilitation for every patient. Professionals need to develop better methods to achieve individual precision in rehabilitations. Screening methods evaluating socioeconomic, psychosocial and physical factors might help health care professionals to individualise the treatment and thereby improving functions and work ability [51, 52]. A limitation in the study is that we have no knowledge about pain patients not offered MMR and if they were offered other customized pain treatments/rehabilitations. No comparison has been made between PHCCs, which recently started compared to PHCCs with long experience and established routines in the team.

This study focuses on organisational level and on different PHCCs in a region in Sweden. Important limitations are that we have no data on details regarding the provided MMR. Discussions or recommendations regarding MMR taking place between patient and physician and whether the patient is expected to benefit from MMR was not captured. Furthermore, PHCCs internal working methods, staff conditions and teamwork have not been analysed due to lack of data, factors which may also be of importance. Likewise data concerning competence among personnel were not available, which therefore could not be taken in to account in this study, but might be important in future research.

Other health care provider factors such as physicians employed by staffing companies, temporary staff and staff turnover at the PHCCs have not been analyzed. These types of factors might also influence referral rates to MMR, but we had no such data available. However, a prerequisite for being assigned to deliver MMR care in Region Skåne is that all competence requirements and implementation of MMR are full-filled.

Equal care is a national goal in Swedish healthcare and patients in need should be offered MMR regardless of which PHCC they visit. Future analysis can provide more details on to what extent patients attended MMR at the same unit as made the referral and if this affect treatment outcome. The results in this study is based on a total cohort from Region Skåne, which, concerning sociodemographic factors, very much correspond to Sweden as a whole. This might indicate that these results are possible to generalize.

In this study we had an organisational perspective and the aim was to study health care provider factors impact on referring rate to MMR. We had no possibility to analyse separate socioeconomic status among the registered population such as income, unemployment, sick leave or care consumption in different areas in Region Skåne, since no individual level data were available. The final regression analyse showed the complexity between factors impact on referral to MMR, such as socioeconomic status, PHCC location/ community size, and whether or not the PHCC provided their own MMR. The importance of organisational factors is confirmed in several recent studies [18, 47, 49].

The overall aim of the National Rehabilitation Program is to improve work ability among patients with mild to moderate mental disorders or MSD. Factors found to be important predictors of sick leave and disability pension due to MSD are employment and social relations [53], stress and workload [54, 55], lifestyle and work factors [56], emotional stability and personality [57]. Future studies will investigate interactions between patient related factors and organisational factors in order to gain further knowledge on successful rehabilitation and return to work.

## Conclusions

We found that referral rates to MMR were positively associated with PHCCs located in medium and large communities with higher socioeconomic status among the registered population, private PHCCs and PHCCs providing their own MMR. Patients with MSD are thus facing significant inequities and were thus not offered the same opportunities for referrals to rehabilitation regardless of which PHCC they visited.

## Abbreviations

ACG: Adjusted Clinical Groups
CNI: Care Need Index
MMR: Multimodal rehabilitation
MSD: Musculoskeletal disorders
PHC: Primary health care
PHCC: Primary health care centre
